# Evaluation of Orthodontic Mini-Implants’ Stability Based on Insertion and Removal Torques: An Experimental Study

**DOI:** 10.3390/bioengineering12050549

**Published:** 2025-05-20

**Authors:** Primavera Sousa-Santos, Sofia Sousa-Santos, Ana Catarina Oliveira, Cíntia Queirós, Joana Mendes, Carlos Aroso, José Manuel Mendes

**Affiliations:** 1 UNIPRO—Oral Pathology and Rehabilitation Research Unit, University Institute of Health Sciences (IUCS), CESPU, 4585-116 Gandra, Portugal; primavera.santos@iucs.cespu.pt (P.S.-S.); carlos.ribeiro@iucs.cespu.pt (C.A.); jose.mendes@iucs.cespu.pt (J.M.M.); 2 Department of Dental Sciences, University Institute of Health Sciences (IUCS), CESPU, 4585-116 Gandra, Portugal; sofia.sousa.santos@hotmail.com (S.S.-S.); acatarina.oliveira@cespu.pt (A.C.O.); a28163@alunos.cespu.pt (C.Q.)

**Keywords:** mini-implants, mini-screws, orthodontic, stability, torque

## Abstract

Orthodontic mini-implants (MIs) are excellent alternative skeletal anchorage devices. Their stability is important for their survival, requiring appropriate torque application during insertion and removal. Objective: This study aimed to evaluate the influences of the diameter and brand of MIs on their stability by measuring the maximum insertion and removal torques after they had been aged in a pH 7 artificial saliva for 4 weeks at 37 °C. Methods: Forty Ti6Al4V alloy MIs of two different brands and diameters were divided into four groups. They were placed in artificial bone blocks using the NSK^®^ Surgic Pro coupled with a digital torque gauge (Centor Touch Star TH^®^) to measure the maximum insertion and removal torques. Results: After ageing, the Fatscrew (Fts) MIs were more stable when removed than the white brand (WB) MIs. The WB MIs lost stability over time, while the Fts MIs—especially the 2.0 mm ones—maintained good stability. Conclusions: The significant differences between the tested groups, especially the stability observed in the 2.0 mm Fts MIs compared to the other groups, highlight the importance of brand and diameter size in the effectiveness of MIs.

## 1. Introduction

The use of orthodontic mini-implants (MIs) has grown significantly in recent years, with their versatility, minimal surgical invasion and relatively low cost being their main advantages [1,2,3,4]. These devices have been successfully used to close spaces; perform movements of intrusion, extrusion, distalisation and verticalisation of molars; correct vertical problems such as open bites and overbites; and address a growing variety of other anchorage needs. Therefore, a more in-depth understanding of MIs is essential to improve treatment outcomes for patients [4]. Despite successful clinical use, quantitative data on how different diameters and brands behave after ageing is lacking, making further research into MIs essential.

Orthodontic MIs are generally made from a titanium alloy (Ti6Al4V) [5,6,7]. Titanium is a highly biocompatible material that allows for direct bone contact between the endosseous dental implants and the host bone. However, the high degree of osseointegration required for dental implants is not a requirement for orthodontic MIs to function as anchorage devices [8,9,10,11]. The use of these systems is based on their primary stability and not on osseointegration [2,3]. Therefore, it is essential to understand the importance of MIs’ stability and the elements that influence it.

Besides histological assessment, several methods for determining the primary stability of MIs have been developed and tested, such as percussion tests, radiological examination, the Periotest and pull-out tests. Unfortunately, most of these methods provide low-precision results [2,12]. Measurements of insertion and removal torques are the most accurate methods for quantitatively assessing the stability and effectiveness of MIs [2,12,13,14]. An adequate insertion torque is necessary to ensure correct fixation of the MIs in the bone, and a controlled removal torque is essential to avoid complications during their removal [2,15]. Removal torque tests are used as a definitive assessment of MIs’ integration into the MI–bone interface [16]. The stability of MIs is fundamental, as tooth movement depends directly on the anchorage they provide. Significant differences between the insertion and removal torques can indicate instability, compromising their effectiveness. Therefore, choosing MIs with good stability and predictable behaviour is essential for achieving satisfactory results.

The reduced dimensions of MIs allow for their insertion in several areas of the oral cavity, but increase the possibility of deformation and fracture during insertion or removal, leading to their failure [17,18]. To avoid such incidents, MIs of a suitable diameter for the quality of the chosen bone site must be used [5,6]. An increase in diameter can lead to significantly greater insertion torque and load during self-drilling, thereby improving MI stability; however, excessive insertion torque can cause detrimental effects such as bone necrosis and increased bone microdamage, potentially contributing to MI failure [2,6,17,19]. If the aim is to achieve high insertion torques and prevent MI fractures, determining the ideal combination of pre-drilling diameters according to the insertion region and bone quality is required [12].

The aim of this investigation was to assess the influence of the diameter and brand of MIs on their stability by measuring the maximum torque during insertion and removal, after the MIs had been subjected to artificial ageing in saliva under controlled conditions, in order to understand whether the MIs would be stable over time.

## 2. Materials and Methods

### 2.1. Materials

All chemicals and materials were used in compliance with the manufacturers’ standards. This investigation analysed two brands of mini-implants (MIs)—Fatscrew brand (Fts) from Air Orthodontics^®^ (Barcelona, Spain) and a white brand (WB) produced by the Worldtrade-center^®^ (Beijing, China) and marketed by eBay^®^ (San Jose, CA, USA). The Fatscrew (Fts) brand by Air Orthodontics^®^ was selected for this study on the basis of its widespread clinical use. The white brand (WB) produced by Worldtrade-center^®^ represents a white brand marketed online for anyone to purchase. The MIs were inserted into an artificial bone with identical characteristics to the human jawbone—Sawbones^®^ (Sawbone Europa AB, Malmö, Sweden). Afterwards, they were placed in pH 7 artificial saliva for 4 weeks (37 °C).

### 2.2. Methods

To analyse all the selected samples, a standard laboratory protocol was established and implemented at the Oral Rehabilitation and Prosthodontics Research Lab, UNIPRO-Oral Pathology and Rehabilitation Research Unit, University Institute of Health Sciences (IUCS), CESPU, Gandra, Portugal.

#### 2.2.1. Sample Preparation

Forty Ti6Al4V alloy (grade V) self-drilling MIs were used in this investigation—twenty Fts brand MIs from Air Orthodontics^®^ and twenty WB MIs from Worldtrade-center^®^, both with 10 mm lengths. For each brand, ten MIs with a diameter of 1.6 mm and ten with a diameter of 2.0 mm were used. The 40 MIs were divided into four groups of 10 MIs each, with all MIs having only one placement in the artificial bone: Group 1 (10 WB MIs of 1.6 mm Ø), Group 2 (10 WB MIs of 2.0 mm Ø), Group 3 (10 FTs MIs of 1.6 mm Ø), and Group 4 (10 Fts MIs of 2.0 mm Ø).

#### 2.2.2. Elaboration of Artificial Bone Block

The material used in this study for the insertion of the MIs was Sawbones^®^ (Sawbone Europa AB, Malmö, Sweden) artificial bone material, with characteristics identical to the human jawbone. Sawbones’ epoxy formulation is filled with short glass fibres and used to simulate cortical bone for structural testing. This simulation of cortical bone has a density of 2.0 g/cc, a fracture toughness of 6.0 MPa, a tensile strength of 150 MPa, a tensile modulus of 20 GPa, a flexural modulus of 20 GPa, a flexural strength of 225 MPa and hardness close to cadaveric cortical bone. Its colour is grey/green. The cellular, rigid polyurethane foam has larger pores in order to closely resemble cancellous bone. There are various types of density to choose from, and it presents an off-white colour [20].

For a better representation of the human jawbone, a fibre-filled epoxy sheet of the 4th generation with 2 mm thickness was used in the cortical bone layer, with the following specifications: short-fibre filler epoxy, with the direction of the fibre parallel to the width (120 mm), and a density of ±2.5%.

To represent the cancellous bone, a rigid cellular foam block of 20 PCF (0.32 g/cm^3^) with a thickness of 10 mm was used, with the following specifications: cellular, rigid polyurethane foam, with the thickness parallel to the direction of rise, and a density of ±10%.

**Figure 1:**
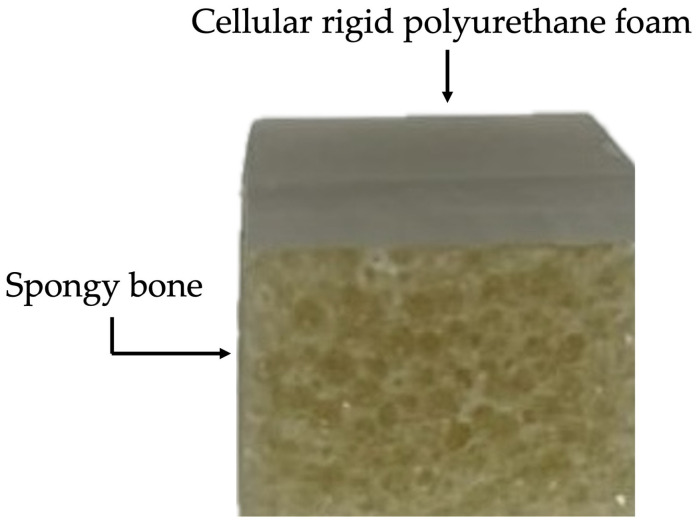
Artificial bone simulation blocks.

The epoxy sheet was attached to the rigid cellular foam block with cyanoacrylate, as indicated by Sawbones^®^. This bone material was divided into 1.5 × 1.5 cm fragments (Figure 1).

#### 2.2.3. Insertion of MIs into Artificial Bone

To ensure uniform placement of the MIs in the artificial bone, the centre of the bone blocks was determined, and the bone blocks were then placed on a support so that they would remain fixed during the MIs’ insertion and removal. The MIs were fixed to an anodised aluminium CNC support (100 × 100 mm), with adjustable claws to keep the 1.5 × 1.5 cm blocks stable during torquing.

The four groups of MIs were inserted into the artificial bone blocks using the NSK^®^ Surgic Pro (NSK. Dental Spain SA, Madrid, Spain), programmed at level 3 with a speed of 200 min^−1^ and a torque of 50 Ncm.

#### 2.2.4. Ageing of MIs in Saliva and Removal from the Artificial Bone

The 40 MIs coupled to the artificial bone were then aged in pH 7 artificial saliva for 4 weeks in a thermostatic incubator at 37 °C, an environment similar to the oral cavity where MIs are used.

The temperature-controlled incubator used was the Memmert^TM^ Peltier-cooled Incubator IPP110 plus (Memmert, Aurich, Germany). The artificial saliva used was based on the Fusayama Meyer formula, an aqueous solution containing 0.4 g/L of NaCl, 0.4 g/L of KCl, 0.795 g/L of CaCl_2_·2H_2_O, 0.005 g/L of Na_2_S·9H_2_O, 0.69 g/L of NaH_2_PO_4_·2H_2_O and 1 g/L of urea [21]. Around 100 mL of the pH 7 artificial saliva was used in each goblet, such that all the artificial bone blocks were completely submerged in the saliva; they were then placed inside the incubator.

After 4 weeks of being submerged in the pH 7 artificial saliva, the MIs were removed from the artificial bone blocks using the NSK^®^ Surgic Pro.

#### 2.2.5. Maximum Insertion and Removal Torque Measurements

During the insertion and removal of the MIs from the artificial bone blocks, the maximum insertion and removal torques were measured continuously using a digital torque gauge attached to the NSK^®^ Surgic Pro.

This research used the Centor Touch Star TH^®^ digital torque gauge manufactured by Andilog Technologies (Vitrolles, France), which is accredited with AB certification and ISO 9001:2015 standards [22].

The data were recorded by connecting the torque gauge attached to the micromotor to a computer. The maximum torque values during insertion and removal were obtained from the maximum peaks on the graphs. The torque force values (Ncm) at insertion and removal were measured. A total of 40 samples were assessed for each torque force, with 10 samples in each group.

#### 2.2.6. Statistical Analysis

The data were analysed with the R software, version 4.3.2 [23]. A three-way repeated-measures (RM) ANOVA was run with one within-group factor (time: insertion vs. removal) and two between-group factors—brand (white label vs. Fts) and diameter (1.6 mm vs. 2.0 mm). A full factorial model was implemented. Next, two-way ANOVAs were run to compare the torque measurements for each brand of MIs with diameters of 1.6 mm (white label vs. Fts) and 2.0 mm (white label vs. Fts). Generalised eta squared (η^2^g) was calculated to measure the effect sizes of the main, 2-way and 3-way effects, according to Cohen’s benchmarks [24] for small (0.01), medium (0.06) and large (0.14) effect sizes.

A Residual QQ plot and Shapiro–Wilk test were used to assess the normality of residuals. Levene’s test was used to test for homoscedasticity of variance. For both tests, the null hypothesis was not rejected when *p* > 0.05. Pairwise comparisons were calculated according to the Bonferroni adjustment. Statistical significance was set at the 5% level.

## 3. Results

Table 1 presents the results for the repeated-measures (RM) ANOVA, with emphasis on the time effect (insertion torque vs. removal torque). Table 2 shows the descriptive statistics for all effects, with the adjusted means and standard errors. The main effects of brand, F (1,9) = 67.13, *p* < 0.001, η^2^g = 0.41, diameter, F (1,9) = 60.10, *p* < 0.001, η^2^g = 0.41, and time, F (1,9) = 211.08, *p* < 0.001, η^2^g = 0.74, were statistically significant with a large effect size. Higher forces (Ncm) were observed for the Fatscrew (Fts) MIs compared to the white-label MIs, for MIs with a diameter of 2.0 mm compared to those with a diameter of 1.6 mm, and for the removal torque compared to the insertion torque. No interaction effect was found for brand vs. diameter (*p* = 0.512) or brand vs. diameter vs. time (*p* = 0.166). The interaction of brand vs. time showed a moderate effect size: F (1,9) = 7.87, *p* = 0.021, η^2^g = 0.07. For the white label brand, the effect size of time (inversion vs. removal) was much higher, F (1,9) = 632.00, *p* < 0.001, η^2^g = 0.85, than that for the Fts brand, F (1,9) = 51.50, *p* < 0.001, η^2^g = 0.59. Significant differences between the insertion and removal torque forces were observed: the torque forces of the Fts MIs with a diameter of 2.0 mm (2.3 Ncm) were lower than those of the Fts MIs with a diameter of 1.6 mm (4.1 Ncm), white-label MIs with a diameter of 1.6 mm (4.4 Ncm) and white-label MIs with a diameter of 2.0 mm (4.5 Ncm).

**Figure 2:**
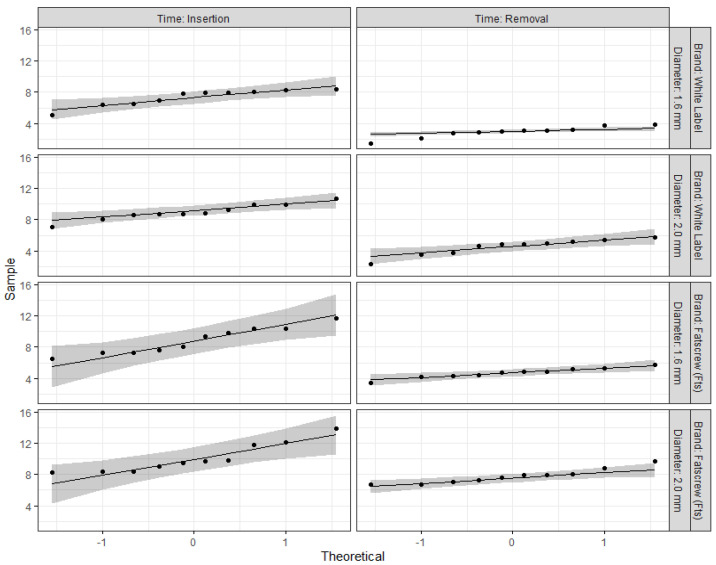
QQ plots for each cell design.

Figure 2 shows the QQ plots for each cell design. Almost all points fell within the normality thresholds. The complementary information provided by the Shapiro–Wilk test did not reject the null hypothesis of normality for any of the cell designs (*p* > 0.05).

**Figure 3:**
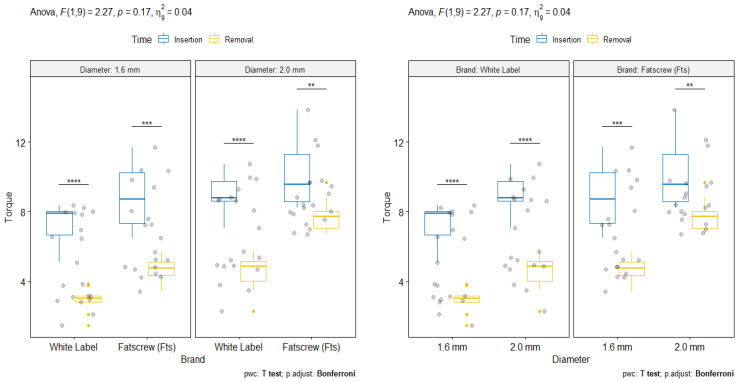
Time (insertion vs. removal) comparisons stratified by brand and diameter. ** *p* < 0.05; *** *p* < 0.01; **** *p* < 0.001.

Figure 3 shows the pairwise comparisons with Bonferroni corrections between insertion and removal torques. As previously noted, all differences were *p* < 0.001 (***), except for the Fts MIs with a diameter of 2.0 mm, where the *p*-value was lower than 0.01 and the boxplots were closer between each other.

**Figure 4:**
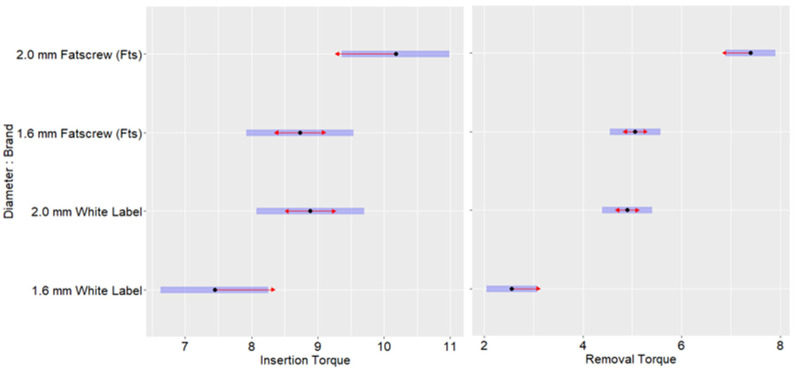
Pairwise comparisons within each torque measurement.

Next, we compared the torque measurements for each brand of MIs with the same diameter: 1.6 mm (white label vs. Fts) or 2.0 mm (white label vs. Fts). Figure 4 shows the pairwise comparisons for the torque measurements, adjusted with the Bonferroni method. Non-overlapping confidence intervals indicated significant results. Regarding the insertion torque of the MIs with a diameter of 1.6 mm or 2.0 mm, no significant differences were found between the white label and Fts. Regarding the removal torque of the MIs with a diameter of 1.6 mm or 2.0 mm, significant differences were found between the white label and Fts. At 2.0 mm, the removal torque of the Fts MIs was significantly higher (*p* < 0.05) than that of the white-label MIs. The same result was observed for the 1.6 mm MIs, with the removal torque of the Fts MIs being significantly higher (*p* < 0.05) than that of the white-label MIs. The adjusted means and standard errors can be found in Table 2.

## 4. Discussion

Skeletal anchorage is essential for a successful orthodontic treatment. In this context, mini-implants (MIs) have been proven to be a valuable solution, reducing the need for patient cooperation and widening treatment options in orthodontics [3,12]. Other skeletal anchorage alternatives, such as dental implants and mini-plates, are also effective, providing stable anchorage for various orthodontic tooth movements. However, MIs stand out due to their lower cost, simpler surgical placement and greater versatility compared to mini-plates and dental implants, thus representing a viable solution to the challenge of achieving dental movement anchorage in orthodontic treatment [1,2,3,4]. Furthermore, MIs offer additional advantages, such as low discomfort for the patient, possibility of immediate loading, and ease of hygiene and removal [3].

Orthodontic MIs can be made from stainless steel or titanium, with the latter having four degrees (I–IV) of decreasing purity, as well as a type V (Ti6Al4V) composed of titanium, 6% aluminium and 4% vanadium, which is most commonly used in the manufacture of MIs [25]. MIs made of Ti6Al4V have better mechanical properties due to their small diameter, resistance to corrosion, and ease of removal and manufacture compared to type IV MIs [2,3,25]. Although their bioactivity is lower than that of pure titanium, which results in a lower rate of osseointegration, the use of these systems is based on primary mechanical stability rather than osseointegration [2,3]. Therefore, 40 Ti6Al4V (grade V) alloy MIs were used in this study, 20 of which were from the Fatscrew brand (Fts) of Air Orthodontic^®^ commercialised by CpmPharma in Portugal, and 20 were white brand (WB) MIs from Wordtrade-center^®^ commercialised on EBay^®^.

Orthodontic MIs range from 6 to 17 mm in length, which is related to their anatomical location, contact with the cortical bone, and the lever arm produced when they are activated [25]. The length of contact with the cortical bone constitutes the main stability factor, while the length of insertion into the cancellous bone has little influence on stability. For this reason, although increasing the length of the MI increases the insertion and removal torques, an insertion length of more than 5 mm does not significantly affect primary stability, as it is inserted into the cancellous bone [25]. Based on this information, we opted for MIs of 10 mm.

In terms of diameter, Walter et al. investigated the effects of MI design characteristics on the mechanical properties of artificial bone, concluding that the outer and inner diameters are the most crucial factors for primary stability at the same implantation depth [26]. The diameter of MIs varies between 1.3 and 2.7 mm, with a larger diameter representing greater stability when heavy forces are applied, and the choice is related to where the MI is implanted as well as its fracture resistance [25]. In our study, for each brand, we used 10 MIs with a diameter of 1.6 mm and another 10 with a diameter of 2.0 mm. The 1.6 mm diameter was chosen because it is a suitable size to be used in all the areas indicated in the oral cavity. The decision to opt for a larger diameter (2.0 mm) was made in order to assess whether this would influence the stability of the MIs.

Many studies have demonstrated the efficacy of MIs, but fracture is one of the risk factors for complications that usually occur during their insertion into and removal from the bone [2,17]. As their diameter decreases, the strength of MIs weakens, leading to potential breakage when the insertion or removal torque exceeds the torsional force [17]. The fracture of MIs during insertion or removal can be a serious problem, inhibiting future tooth movement and, at times, requiring surgical removal [2,17]. Melsen et al. associated a smaller diameter of MIs with a greater possibility of fracture, which occurs more frequently during their removal than during insertion [27]. Fracture generally occurs near the neck of the MI and the presence of holes can further weaken the device [2,27]. In this sense, it is extremely important to assess the influence of diameter on the effectiveness of MIs.

Given the difficulty of working with bone in vivo, in this study, the MIs were inserted into polymeric samples of different densities, known as Sawbones^®^, which simulated the structure of trabecular bone [18]. Due to the complications involved in handling trabecular bone, synthetic polyurethane foams are widely used as alternative materials to this type of bone in diverse biomechanical tests, as they have a similar cellular structure and equivalent mechanical characteristics [28]. Sawbones^®^ consists of polymeric materials and is used as a standard material for carrying out mechanical tests in accordance with the American Society for Testing and Materials (ASTM) (ASTM F-1839-08 (2021)) [29]. Although artificial foams have limitations and do not fully represent real human jawbone, they are widely used in biomechanical tests, simulations and evaluation of dental implants [18,30,31]. To represent the human jawbone, it is necessary to have two artificial bones with different characteristics to represent the cancellous bone and the cortical bone, helping to optimise the similarities with the human bone. In our study, we used a 2 mm thick sheet made of fibre-filled epoxy to represent the cortical bone and a 10 mm thick rigid block made of 20 PCF (0.32 g/cm^3^) cellular foam to represent the cancellous bone, a technique we had already carried out in a previous study where we evaluated the influence of the MI diameter on primary stability [32]. All MIs were placed in a block of Sawbones^®^ artificial bone.

An MI’s primary stability is determined based on its mechanical retention in the bone, which depends on the bone properties, MI design engineering and placement technique [15,33]. In turn, an MI’s secondary stability is determined by its biological union with the surrounding bone, which is affected by its surface, characteristics, bone turnover and the mechanical system used [15]. According to Proffit, clinical stability corresponds to the sum of primary and secondary stability, decreasing up to 2 weeks and reaching the maximum value 4 weeks after the insertion of the MI into the bone [15]. Considering this, we decided to measure the MIs’ removal torque from the bone 4 weeks after being submerged in artificial saliva in a thermostatic incubator at 37 °C that simulated the environment of the oral cavity in which MIs are used. Sufficient primary stability, as measured based on the insertion torque, seems to play an important role in MIs’ survival rate during orthodontic treatment, with the maximum insertion torque value often used to quantify the initial stability [1,12,17,31,34]. A high insertion torque can indicate a mechanically stable MI. A systematic review concluded that a wide range of insertion torque values can achieve high MI success rates, but excessive insertion torque can cause detrimental effects such as bone necrosis and increased bone microdamage [19]. Microdamage initiates rapid bone remodelling and healing but, if accumulated, bone mechanical properties decrease, potentially contributing to MI failure [2,17,19]. If the aim is to achieve high insertion torques while preventing possible MI fractures, one must determine the ideal combination of pre-drilling diameters according to the insertion region and bone quality [12]. Determining the maximum insertion torque peak of an MI represents a non-destructive method of measuring its stability [31]. An insertion torque of 5–10 Ncm is recommended for MIs with a diameter of 1.6 mm to minimise any risk of failure, as higher values can cause them to fracture [1,12]. None of the MIs used in our study had insertion torque values higher than 10 Ncm, ensuring that the MIs from both brands, Fts and WB, were safe to use.

Elias et al. found that, when comparing two types of MIs from the same manufacturer with different diameters, the MIs with a larger diameter had a greater insertion torque, as the insertion torque is proportional to the area of contact between the MI and the bone [3]. These results were similar to those obtained in our study, with the 2.0 mm MIs showing higher insertion torque values for both the Fts and WB brands. Additionally, these authors obtained an average insertion torque of 9.6 Ncm in rabbit cortical bone and 12.6 Ncm in bovine cortical bone for 1.5 mm MIs [3], which were also similar to the values obtained in our study. Their study also found that MIs with a diameter of 2.0 mm had an average insertion torque of 23.2 Ncm when inserted into bovine cortical bone [3], which was higher than the values obtained in our study. However, this difference can be explained by the difference in the type of bone used in each study. Brown et al. also obtained an average maximum insertion torque value of 11.0 Ncm [17]. Motoyoshi et al. obtained insertion torque values that ranged from 7.2 to 13.5 Ncm in adults [35] and from 7.6 to 9.2 Ncm in adolescents [36]. In another study by Motoyoshi et al., good success rates were obtained when placing 1.6 mm diameter MIs with an insertion torque of 8 to 10 Ncm in cortical bone thicker than 1 mm [37]. The same results were achieved in our investigation.

In our study, insertion torque values similar to those reported in previous studies were obtained for both the WB and Fts MIs. No significant differences were found in the insertion torque values between the two brands. Although the insertion torque values of the 2.0 mm MIs were always higher than those of the 1.6 mm diameter MIs, no significant differences were found with respect to diameter between the WB or Fts MIs.

During bone healing after MI insertion, new bone is formed and remodelled around it [14,17]. The proportion of bone–implant contact is crucial to its effectiveness, with only 5% of this contact being necessary to resist orthodontic loads [8]. The removal torque of MIs, ranging from 4 to 16 Ncm, reflects their anchoring capacity and is affected by the formation of fibrous tissue around the MIs’ threads [13,14]. This torque increases with lamellar compaction during bone healing, reaching a higher level approximately 4 weeks after insertion [15]. There are few studies evaluating the maximum removal torque. Generally, the removal torques reported in short-term studies are much lower than the insertion torques; however, it is necessary to have a follow-up period of 4 weeks, during which time removal torques may undergo alterations [2,3]. With regard to the removal torque values in our study, after ageing in artificial saliva for 4 weeks, there were significant differences between the two brands. For both the 2.0 mm and 1.6 mm diameters, the removal torque of the Fts MIs was significantly higher than that of the WB MIs. These results were comparable to the values reported in the study by Brown et al. [17]. When evaluating the stability of the MIs, after ageing in artificial saliva for 4 weeks, the removal torque obtained was significantly lower than the insertion torque, except in G4 (2.0 mm Fts MIs), which was similar to the study by Brown et al. [17]. The difference between the insertion and removal torques of the 2.0 mm Fts MIs was the lowest in our study (2.3 Ncm), indicating that these MIs achieved better fixation rates within the bone. The greatest differences between the insertion and removal torques were found for the 2.0 mm WB MIs (4.5 Ncm) and the 1.6 mm WB MIs (4.4 Ncm). The Fatscrew mini-implants demonstrated higher stability, likely due to their tapered design and deeper double-threaded configuration, which promotes stronger mechanical interlocking with the surrounding substrate. Conversely, the White Brand mini-implants exhibited a less aggressive thread profile and a more cylindrical body, which may explain their lower performance in terms of stability.

Wilmes et al. recommend limiting the insertion torque to 20 Ncm, due to the fractures observed in MIs with torques above 23 Ncm, in order to avoid fracturing them [12]. MI fracture is a particular concern in areas such as the second pre-molar or second molar regions, where proximity to the inferior alveolar nerve canal complicates the procedures to remove a fractured MI [1]. In our study, the insertion and removal torque values were less than 20 Ncm, indicating that they are safe and unlikely to cause damage.

Although our study simulates various parameters, it is important to recognise that any study design that aims to reproduce the complex biomechanics of the oral environment has certain limitations, and the results must be carefully interpreted. Clinical conditions such as periodontitis or gastroesophageal reflux disease (GERD) can create a more acidic oral environment, increasing chemical reactivity and potentially causing modifications to the MIs used [21].

## 5. Conclusions

Based on the results obtained and according to the methodology described in this study, the following conclusions can be drawn:

No significant interaction was found between diameter within each brand, but the 2.0 mm mini-implants (MIs) showed higher insertion torque values than the 1.6 mm ones, suggesting an important relationship between diameter and torque force.

No significant differences were found with regard to the insertion torques between the two brands.

After ageing in artificial saliva for 4 weeks, there were significant differences between the two brands. The removal torque values of the Fatscrew (Fts) MIs were significantly higher than those of the white brand (WB) MIs for both the 2.0 mm and 1.6 mm diameters.

The WB MIs showed a consistent tendency towards less stability, with significant differences between the insertion and removal torque values, indicating a considerable loss of stability over time.

The Fts MIs with a diameter of 2.0 mm showed relatively greater stability, with smaller differences between the insertion and removal torque values, indicating a more consistent and reliable performance over time.

The results highlight the significant influence of brand and diameter on the torque values of the insertion and removal forces of the MIs.

## Figures and Tables

**Table 1:** Repeated-measures (RM) ANOVA effects.

	F-Test_(1,9)_	*p*-Value	η^2^_g_
Three-way effects			
Brand	67.13	<0.001 ***	0.41
Diameter	60.10	<0.001 ***	0.41
Time	211.08	<0.001 ***	0.74
Brand vs. diameter	0.47	0.512	0.011
Brand vs. time	7.87	0.021 *	0.07
Diameter vs. time	2.72	0.133	0.04
Brand vs. diameter vs. time	2.27	0.166	0.04
Two-way effects (stratified by brand)			
Brand: White label			
Diameter	13.90	0.005 **	0.44
Time	632.00	<0.001 ***	0.85
Diameter vs. time	0.01	0.936	0.00
Brand: Fatscrew (Fts)			
Diameter	19.10	0.002 **	0.40
Time	51.50	<0.001 ***	0.59
Diameter vs. time	3.44	0.097	0.11
Simple effects (stratified by brand: diameter)			
White label: 1.6 mm	131.00	<0.001 ***	0.87
White label: 2.0 mm	181.00	<0.001 ***	0.84
Fts: 1.6 mm	40.60	<0.001 ***	0.74
Fts: 2.0 mm	11.20	0.009 **	0.40

η^2^g, generalised eta squared; * *p* < 0.05; ** *p* < 0.01; *** *p* < 0.001.

**Table 2:** Adjusted marginal means and pairwise comparisons for three-way RM ANOVA.

	Insertion Torque (Ncm)	Removal Torque (Ncm)
Brand		
White label	8.16 (0.33)	3.73 (0.20)
Fatscrew (Fts)	9.45 (0.33)	6.23 (0.20)
Overall	8.81 (0.23)	4.98 (0.13)
Diameter		
1.6 mm	8.09 (0.33)	3.81 (0.19)
2.0 mm	9.53 (0.33)	6.15 (0.19)
Overall	8.81 (0.23)	4.98 (0.13)
Brand: White label		
Diameter: 1.6 mm	7.34 (0.47)	2.93 (0.27)
Diameter: 2.0 mm	8.99 (0.47)	4.52 (0.27)
Brand: Fts		
Diameter: 1.6 mm	8.83 (0.47)	4.69 (0.27)
Diameter: 2.0 mm	10.07 (0.47)	7.77 (0.27)

## Data Availability

The original contributions presented in the study are included in the article, further inquiries can be directed to the corresponding author.
